# Current status and challenges in the assessment of antibiotic allergy information in electronic health records: a retrospective observational study of perioperative patients

**DOI:** 10.1017/ash.2026.10777

**Published:** 2026-07-07

**Authors:** Yui Enatsu, Yasuaki Tagashira, Koh Okamoto, Akane Takamatsu, Yoshiaki Gu

**Affiliations:** 1 Department of Infection Prevention and Control, Institute of Science Tokyo Hospital, Tokyo, Japan; 2 Department of Pharmacy, Graduate School of Medical and Dental Sciences, Institute of Science Tokyo, Tokyo, Japan; 3 Department of Infectious Diseases, Graduate School of Medical and Dental Sciences, Institute of Science Tokyo, Tokyo, Japan; 4 Center for Infectious Disease Education and Analysis (TCIDEA), Institute of Science Tokyo, Tokyo, Japan

## Abstract

**Background:**

Inappropriate antibiotic allergy labeling often leads to the unnecessary avoidance of first-line therapies. The present study aimed to evaluate the current status of antibiotic allergy documentation and its assessment in patients undergoing surgery for which cefazolin was the recommended first-line prophylaxis in a Japanese hospital.

**Methods:**

This retrospective observational study was conducted at a university hospital in Tokyo from 2021 to 2023; included patients with a history of antimicrobial allergy who underwent surgery for which cefazolin was recommended; and assessed patient demographics, details of the allergies in electronic health records (EHR), and perioperatively administered antimicrobial agents.

**Results:**

Of 2,402 eligible patients, 243 (10.1%) had a registered antimicrobial allergy. The drug classes most frequently recorded in the EHR were cephalosporins (25.0%) and penicillins (24.0%). Documentation of allergy assessments by a physician (9.0%) or specialist (7.0%) was rare. Although 51.9% of the labeled patients had received cefazolin, non-first-line agents had also been frequently administered. Notably, clindamycin had been administered to 30% of the cases and had been widely used even among patients with only a penicillin allergy label despite the low risk of cross-reactivity.

**Conclusions:**

Antibiotic allergy labels in the EHR were often incomplete, infrequently assessed, and associated with substantial avoidance of first-line prophylaxis. Redesigning the EHR format to allow allergies to be distinguished from adverse events while retaining the standard evaluation pathways is essential to optimizing perioperative antimicrobial stewardship.

## Introduction

Preventing surgical site infections (SSIs) depends on the timely administration of effective, perioperative, antimicrobial prophylaxis. Cefazolin is widely recommended as first-line prophylaxis for many clean and clean-contaminated procedures because of its activity spectrum, safety profile, and cost.^1,2^ However, patients with a beta-lactam antibiotic allergy label often receive alternative, prophylactic agents, such as clindamycin and vancomycin.^3^ Inappropriate labeling is associated with the avoidance of first-line beta-lactam agents for treatment and prophylaxis, which increases the risk of SSI,^3^ rate of infections by antimicrobial-resistant organisms and *C. difficile*,^4^ duration of hospitalization, and healthcare costs.^5^ For these reasons, appropriately assessing patients’ information about antibiotic allergy is crucial to effective antimicrobial stewardship.^5,6^

Antibiotic allergy labels are commonly recorded in electronic health records (EHR). When patients self-report an antibiotic allergy, this information is typically entered into their EHR by a physician, nurse or pharmacist. However, this information is often documented without sufficiently evaluating the specific symptoms, timing or clinical course. Indeed, many of the labels are in fact of nonallergic adverse drug reactions or historical events that are no longer clinically relevant.^7,8^ The clinical impact of an allergy label depends not only on its availability, but also on the thoroughness of the information documented, such as the name of the causative agent, reaction phenotype, timing, recency, and whether the label has been assessed or re-evaluated.^7,8^ In the perioperative setting, incomplete or ambiguous information about patients’ allergies may lead to a precautionary avoidance of certain agents as first-line prophylaxis.^3^

Evidence of the epidemiology and downstream effects of the beta-lactam allergy label is generally quite robust in the Western countries but remains limited in Asia, where the reported prevalence and assessment methods vary.^9^ Given the differences in patterns of antimicrobial use, EHR systems, and access to allergologists, data are needed from each relevant region to inform a tailored approach to perioperative prophylaxis and antimicrobial stewardship. In Japan, there are scant data on the quality of antibiotic allergy-related documentation in EHR and real-world methods of perioperative assessment. In particular, it remains unclear how often the key elements of the documentation are missing, how frequently clinicians document assessment results or seek evaluation by a specialist, and to what extent these factors are associated with the use of non-first-line, prophylactic regimens.

The present study therefore had the three-fold aim of characterizing the completeness of antibiotic allergy labels in the EHR of patients undergoing surgery for which cefazolin was recommended as first-line prophylaxis; quantifying the frequency and content of documented clinical assessments and specialist evaluations of those labels; and describing the perioperative, prophylactic regimens actually administered (cefazolin vs non-first-line alternatives) in relation to the recorded labels.

## Methods

### Study setting and patient selection

This retrospective observational study was conducted at the Institute of Science Tokyo Hospital (ISTH, formerly Tokyo Medical and Dental University Hospital), an 813-bed tertiary teaching hospital with 19 surgical departments located in Tokyo, Japan. The study period was April 2021 through March 2023.

### Patient identification and eligibility criteria

This study included adult patients (aged ≥ 18 yr) who had undergone surgery in any one of the following surgical departments: orthopedic, obstetrics and gynecology, peripheral vascular surgery, thoracic surgery, breast surgery, cardiac surgery, general surgery, and cardiology. These departments were chosen for analysis because they administered a cefazolin-based prophylaxis as the first-line regimen for most procedures in accordance with departmental protocol and accounted for most of the eligible surgeries during the study period. Cardiology was included because certain invasive procedures at the study center are managed within the cardiology department and follow the same perioperative prophylaxis workflow. Eligible cases were identified by linking operating room and procedural scheduling records with those of perioperative medication administration to capture prophylactic antibiotic use. These cases were then cross-referenced with a dedicated EHR allergy module to identify patients with antibiotic allergy label ≥1. The exclusion criteria were age <18 years and change in the recommended prophylactic regimen due to intraoperative conversion or procedural modification resulting in the exclusion of cefazolin as the recommended first-line agent.

### Recommendations for institutional prophylaxis

Procedures for which cefazolin was recommended as first-line prophylaxis were those described as such by the Japanese guidelines for perioperative antimicrobial prophylaxis^10^ and the corresponding, institutional prophylaxis protocol. The latter dictates the use of clindamycin or vancomycin as an alternative, prophylactic regimen when a beta-lactam allergy label is present.

### Data elements and definitions

The study center uses a commercial EHR system (HOPE/EGMAIN-GX, Fujitsu, Japan). Antibiotic allergy labels are entered into a dedicated allergy module that records the causative drug (selected from a list of drug names) and a free-text description of the reaction details. Structured fields for timing and severity are available but not mandatory, and the module does not distinguish immune-mediated allergies from nonallergic adverse drug reactions at the time of entry.

The term, “antibiotic allergy label” was used to refer to any antibiotic agent recorded in the allergy module regardless of whether the recorded event represented a true, immune-mediated allergy or a nonallergic adverse reaction. The following information was extracted from each label: (1) causative antibiotic agent or drug class; (2) symptoms and findings; (3) timing of symptom onset (categorized as <10 yr, ≥10 yr or unknown); (4) evidence of clinical assessment by a physician (ie, any documentation indicating a review or reinterpretation of the label); (5) record of a specialist’s evaluation (consultation with a dermatologist or infectious diseases specialist to evaluate the label, including documentation of assessment items, whenever available); and (6) perioperatively administered antibiotic agents.

Symptoms and findings were categorized a priori into clinically meaningful groups, and eligibility for re-administration was assessed on the basis of the documented symptoms and findings: (1) not eligible, reactions indicating a severe, immune-mediated mechanism (eg, an immediate hypersensitivity reaction to a cephalosporin or penicillin, or a severe cutaneous adverse reaction); (2) eligible, a reaction attributable only to antibiotics other than cephalosporins and penicillins, or nonspecific intolerance to cephalosporins or penicillins unlikely to represent a severe, immune-mediated mechanism; and (3) indeterminate, cases in which the mechanism or severity could not be determined from the medical record alone. Table 1 shows the detailed mapping by category.

**Table 1.** Registered symptoms, classification of antimicrobial allergies and adverse effects, and criteria for re-administration

Category	Documented symptoms		
	Not eligible	Eligible	Indeterminate
Systemic symptoms	Anaphylaxis	Fatigue	Fever
Dermatological symptoms	Drug eruption, urticaria		Erythema, itching, suspected photosensitivity
Respiratory symptoms/findings	Dyspnea, asthmatic symptoms		Suspected drug-induced pneumonitis
Gastrointestinal symptoms		Nausea, diarrhea, enteritis	Gastrointestinal bleeding
Neurological symptoms		Unsteadiness, dizziness	Disturbance of consciousness
Head and neck symptoms/findings	Pharyngeal edema, facial swelling		Ocular hyperemia
Renal findings			Interstitial nephritis
Hepatic findings			Abnormality of liver function
Blood pressure-related findings	Hypotension, blood pressure instability		
Hematological findings			Leukopenia, neutropenia
Unclassifiable symptoms/findings			Unknown symptoms, no recollection by patient

### Outcomes

The primary outcomes were the proportions of labels documenting the reaction phenotype and documenting timing or recency. Secondary outcomes were the proportions of labels documenting clinical assessment, the proportion of patients receiving specialist evaluation, and labeled patients receiving perioperative cefazolin. Clinical outcomes included surgical site infection (SSI) within 30 days, *Clostridioides difficile* infection (CDI) within 90 days, and perioperative adverse drug reactions.

To assess the potential effect of labeling on the choice of prophylactic agent, the use of non-first-line prophylaxis was evaluated in patients with labels limited to (1) cephalosporins, (2) penicillins, and (3) both cephalosporins and penicillins.

### Statistical analysis

Descriptive statistics were used for analysis. Categorical variables were expressed as a number and a percentage, and continuous variables were expressed as the median and a range. All analyses were performed at the patient and label levels, as appropriate. This study was approved by the Institutional Review Board of Institute of Science Tokyo Hospital (approval number M2024-035). The requirement for patient consent was waived in accordance with the Declaration of Helsinki because the study was retrospective. This study was performed in accordance with the Strengthening the Reporting of Observational Studies in Epidemiology guidelines.

## Results

### Patient characteristics

During the study period, 2,402 adult patients underwent a procedure in one of the eight included departments, of whom 253 (10.5%) had antimicrobial allergy labels. Of the 2,402, 1,981 (82.5%) underwent a procedure for which the institutional protocol recommended cefazolin as first-line perioperative prophylaxis. After excluding patients aged <18 years and those whose regimen was changed due to intraoperative conversion or substantial procedural modification, 243 patients (10.1%) remained for the final analysis. The median age was 65 years (range: 19–93), and 174 (71.6%) were female. The most common underlying conditions were solid malignancies (21.4%), autoimmune diseases (17.7%), and chronic lung diseases (16.0%) (Table 2).

**Table 2.** Demographic data of patients with registered antimicrobial allergies

Variables	Total (N = 243)		Cefazolin group (N = 131)		Non-cefazolin group (N = 112)	
Patient characteristics						
Age, years (min-max)	65	(19–93)	64	(19–93)	66	(19–87)
Female sex	174	(71.6)	92	(52.9)	82	(47.1)
Solid malignant tumor	52	(21.4)	25	(48.1)	27	(51.9)
Connective tissue disease	42	(17.7)	21	(50.0)	21	(50.0)
Chronic pulmonary disease	39	(16.0)	24	(61.5)	15	(38.5)
Diabetes mellitus	35	(14.4)	17	(48.6)	18	(51.4)
History of immunosuppressant use	25	(10.3)	10	(40.0)	15	(60.0)
Chronic kidney disease	23	(9.5)	14	(60.9)	9	(39.1)
History of steroid administration > 5 mg/day	22	(9.1)	7	(31.8)	15	(68.2)
Cerebrovascular disease	15	(6.2)	7	(46.7)	8	(53.3)
Hematological malignancy	11	(4.5)	5	(45.5)	6	(54.5)
Chronic heart failure	6	(2.5)	3	(50.0)	3	(50.0)
Allergic skin disease	6	(2.5)	2	(33.3)	4	(66.7)
Chronic liver disease	2	(0.8)	1	(50.0)	1	(50.0)
Dementia	0	(0.0)	0	(0.0)	0	(0.0)
Orthopedic surgery	111	(45.7)	66	(59.5)	45	(40.5)
Obstetrics and gynecology	35	(14.4)	18	(51.4)	17	(48.6)
Peripheral vascular surgery	29	(11.9)	11	(37.9)	18	(62.1)
Thoracic surgery	22	(9.1)	9	(40.9)	13	(59.1)
Breast surgery	18	(7.4)	9	(50.0)	9	(50.0)
Cardiac surgery	17	(7.0)	14	(82.4)	3	(17.6)
General surgery	7	(2.9)	3	(42.9)	4	(57.1)
Cardiology	4	(1.6)	1	(25.0)	3	(75.0)

### Details of antibiotic allergies

In total, 312 antimicrobial agents were registered in the 243 patients, with the median being one registered agent per patient (range: 1–8) (Table 3). The most frequently recorded antibiotic classes were cephalosporins (78/312; 25.0%) and penicillins (75/312; 24.0%), followed by fluoroquinolones (40/312; 12.8%). Third-generation agents accounted for the largest proportion of the cephalosporin labels (35/78; 44.9%), followed by first-generation agents (29/78; 37.1%) and fourth-generation agents (5/78; 6.4%); the remainder consisted of other cephalosporins.

In total, 347 symptoms were recorded for the 312 registered antimicrobial agents (Table 4). Dermatological symptoms were the most common (171 events, 49.3%), with reactions suggesting a severe immune-mediated mechanism, including drug eruption and urticaria, in 11 cases (6.4%); these were followed by systemic (35; 10.1%) and gastrointestinal symptoms (35; 10.1%). A substantial proportion lacked details (eg, “unknown” or “no recollection”; 57 registrations, 16.7%), hampering risk stratification. Anaphylaxis was documented in 17 patients (5.5%). Documentation of timing and recency was frequently incomplete: symptom onset was not documented for 233/312 labels (74.7%), and where available, was within 10 years for 41/312 (13.1%) and ≥10 years for 38/312 (12.2%).

**Table 3.** 312 antimicrobial agents registered as those of an antimicrobial allergy (multiple entries allowed) in the 243 patients

Category of antimicrobial agent	N	(%)
Cephalosporins	78	(25.0)
Penicillins	75	(24.0)
Fluoroquinolones	40	(12.8)
Macrolides	24	(7.7)
Trimethoprim-sulfamethoxazole	24	(7.7)
Tetracyclines	23	(7.4)
Lincosamides	8	(2.6)
Glycopeptides	7	(2.2)
Antituberculosis drugs	6	(1.9)
Chloramphenicol	5	(1.6)
Unknown	15	(4.8)
Other^*^	7	(2.2)

* Other categories included carbapenems in 3 patients (1.0%); fosfomycin in 2 patients (0.6%); aminoglycosides and nitroimidazoles in 1 patient each (0.3%).

**Table 4.** 347 Symptoms and findings registered as an antimicrobial allergy (multiple entries allowed) in the 243 patients

Symptoms and findings	Frequency	
Dermatological symptoms	171	(49.3)
Drug eruption, urticaria	11	(6.4)
Erythema, itching, suspected photosensitivity	160	(93.6)
Unclassifiable symptoms/findings	58	(16.7)
Systemic symptoms	35	(10.1)
Gastrointestinal symptoms	35	(10.1)
Hepatic findings	11	(3.2)
Hematological findings	10	(2.9)
Neurological symptoms	8	(2.3)
Head and neck symptoms/findings	8	(2.3)
Respiratory symptoms/findings	6	(1.7)
Blood pressure-related findings	3	(0.9)
Renal findings	2	(0.6)

Note: Data are presented as a number (%) unless otherwise specified.

Clinical assessment by a physician was documented in only 28/312 labels (9.0%). Assessments by a specialist were uncommon: documentation of dermatological consultations for the pre or postoperative assessment of antibiotic allergies was available for 15/243 patients (6.2%), and documentation of consultations with an infectious diseases specialist was available for 2/243 patients (0.8%).

Among the patients with a registered antimicrobial allergy, the most frequently administered, perioperative antimicrobial agent was cefazolin (126 patients; 51.9%), followed by clindamycin (73 patients; 30.0%) and fosfomycin (12 patients; 4.9%) (Table 5). Non-first-line prophylaxis was frequently used even in the patients with only a penicillin label. Among 69 patients with only a cephalosporin label, non-cefazolin prophylaxis was administered to 50 patients (72.5%). Of 62 patients with only a penicillin label, non-cefazolin prophylaxis was administered to 46 patients (74.2%). In seven patients with a label limited to a penicillin *and* a cephalosporin, non-cefazolin prophylaxis was administered to six patients (85.7%). (Table 6). In this study, patients with a history of immunosuppressant use, history of steroid administration >5 mg/day, and allergic skin disease were more common in the group that did not receive cefazolin for perioperative prophylaxis (40.0% vs 60.0%, 31.8% vs 68.2%, and 33.3% vs 66.7%). Reactions to each antimicrobial agent recorded in the 243 patients were assessed using prespecified criteria for re-administration. Overall, 106 patients (43.6%) were classified as eligible, 24 (9.9%) as not eligible, and 113 (46.5%) as indeterminate. As clinical outcomes, we observed no SSI or CDI. However, perioperative adverse drug reactions were observed in 7 patients; dermatological symptoms in 5 patients (71.4%), followed by blood pressure-related events in 1 patient (14.3%) and hematological events in 1 patient (14.3%).

**Table 5.** Antimicrobial agents actually administered during the perioperative period in patients with a documented allergy (N = 243)

Antimicrobial regimen	N	(%)
Cefazolin	126	(51.9)
Clindamycin	73	(30.0)
Fosfomycin	12	(4.9)
Ampicillin/sulbactam	6	(2.5)
Piperacillin/tazobactam	4	(1.6)
Other^*^	22	(9.1)

* Other regimens included levofloxacin in 3 patients (1.2%); cefazolin + clindamycin; cefazolin + vancomycin in 2 patients each (0.8%); vancomycin; ceftriaxone; cefepime; meropenem; ampicillin; clarithromycin; cefmetazole; piperacillin; aztreonam + vancomycin; vancomycin + meropenem; ceftriaxone + vancomycin; cefepime + vancomycin; daptomycin + cefazolin; clindamycin + gentamicin; and none in 1 patient each (0.4%).

**Table 6.** Alternative antimicrobial agents administered perioperatively in patients with a registered cephalosporin or penicillin allergy

Registered allergy	Alternative agent	N (%)
**Cephalosporins only** (n = 69)	Received a non-cefazolin agent (n = 50)	
	Clindamycin	36 (72.0)
	Fosfomycin	4 (8.0)
	Piperacillin/tazobactam	3 (6.0)
	Ampicillin/sulbactam	2 (4.0)
	Other^*^	5 (10.0)
**Penicillins only** (n = 62)	Received a non-cefazolin agent (n = 46)	
	Clindamycin	33 (71.7)
	Fosfomycin	7 (15.2)
	Ampicillin/sulbactam	2 (4.3)
	Levofloxacin	2 (4.3)
	Cefmetazole	1 (2.2)
	None	1 (2.2)
**Cephalosporins + penicillins** (n = 7)	Received a non-cefazolin agent (n = 6)	
	Clindamycin	4 (66.7)
	Clarithromycin	1 (16.7)
	Fosfomycin	1 (16.7)

* Other agents included levofloxacin; ampicillin; cefepime; ceftriaxone + vancomycin; aztreonam + vancomycin in 1 patient each (2.0%).

## Discussion

This retrospective, single-center study evaluated the prevalence and characteristics of antibiotic allergy labels in the EHR, as well as the extent of the clinical assessment of these labels in patients in a Japanese university hospital undergoing a procedure for which cefazolin was recommended as the first-line perioperative prophylactic agent. Approximately 10% of the eligible patients had an antibiotic allergy label. Cephalosporins were the most frequently labeled class, followed by penicillins and fluoroquinolones. Among the patients with an antibiotic allergy label, roughly half received a cefazolin; however, an alternative agent was used in the remaining cohort. Clindamycin was the most common alternative, and cefazolin was often avoided even in patients labeled as having an allergy only to penicillin. The quality of the documentation was suboptimal, and both primary-team assessment and specialist consultation for allergy evaluation in this group were infrequent.

The prevalence of antibiotic allergy labels in this study (10.1%) was comparable to previous reports of self-reported penicillin allergy in other countries (approximately 8%)^11^ and the findings of a previous, Japanese, single-center study of patients who had received infectious diseases consultation (approximately 13%).^12^ The predominance of cephalosporin labels was also consistent with Japanese data on pregnant patients.^13^

Our findings highlighted the frequent use of clindamycin as an alternative to cefazolin for perioperative prophylaxis. The Japanese guidelines recommend clindamycin or vancomycin as alternatives for patients with a beta-lactam allergy label.^10^ In our cohort, clindamycin was administered to approximately 70% of patients with a penicillin and/or cephalosporin allergy label, suggesting it is widely perceived as a standard substitute with perioperative timing and workflow similar to cefazolin. However, current evidence indicates that cross-reactivity between penicillins and cephalosporins is substantially lower than historically assumed, especially when side-chain structures differ.^14^ Cefazolin in particular is structurally distinct from many other beta-lactam agents and is considered to have a low risk of cross-reactivity based on side-chain considerations.^15^ Importantly, the use of non-first-line alternatives (including clindamycin and/or vancomycin) in patients labeled penicillin-allergic has been associated with a higher rate of SSI.^16^ When eligibility for re-administration was assessed from the recorded symptoms, approximately 40% of cases were likely eligible. These data suggest that systems enabling appropriate identification of patients who can safely receive cefazolin could improve both antimicrobial stewardship and patient outcomes. Because the cohort also included patients with allergies to antimicrobials other than beta-lactams, physicians may have individually reviewed the allergy history before selecting cefazolin.

This study also identified limitations in EHR functionality and workflow: allergy labels and nonallergic adverse drug reactions were not clearly distinguishable in the EHR, raising the possibility that nonallergic events were recorded as an allergy, leading to unnecessary avoidance of first-line agents. In addition, key elements required for risk stratification—the identity of the causative agent, details of the reaction phenotype, and timing—were frequently missing, making subsequent clinical assessment challenging, as also noted in previous studies.^7,8^ These findings underscore the need to improve both EHR design and local documentation practices, such as standardizing entry of causative agents, recording reaction details, and establishing a chronology of events. Patients with a history of immunosuppressant use, steroid administration >5 mg/day, or allergic skin disease were significantly more common in the group that did not receive cefazolin. Atopic predisposition has been reported as a predictor of hypersensitivity reactions to beta-lactam antimicrobials, and clinicians may have avoided cefazolin in light of these findings; however, the extent to which they are recognized in routine clinical practice remains unclear.^17^ Additionally, allergy labels were also more common in women than in men; previous studies have similarly reported that antimicrobial allergy labels, particularly beta-lactam labels, are more frequent among female patients, which may have contributed to the higher proportion of women in this cohort.^18^

Recent studies have reported that structured assessment and de-labeling interventions led by trained clinicians (including pharmacists and physicians) can improve the quality of allergy documentation and increase the appropriate use of beta-lactam agents.^19^ The Centers for Disease Control and Prevention’s Global Antibiotic Stewardship Evaluation Tool also positions antibiotic allergy assessment as an important component of inpatient antimicrobial stewardship.^20^ Establishing a feasible, context-appropriate evaluation pathway, potentially involving an allergy assessment team, may therefore be a key step in improving the accuracy of antibiotic allergy information and optimizing perioperative prophylaxis. In particular, preoperative assessment clinics provide an effective opportunity to reassess and remove inappropriate penicillin allergy labels before surgery, and pharmacist-led interventions have been reported to enable de-labeling in a larger number of patients.^21^

This study has several limitations. First, it was a retrospective analysis at a single academic hospital; thus, its findings may not be generalizable to other settings in Japan. Second, the cohort was restricted to patients undergoing a procedure for which cefazolin was recommended as first-line prophylaxis; patterns may differ for other procedures and specialties. Third, because the EHR allergy module could not distinguish allergic reactions from other adverse events, the proportion of genuine, immunologically mediated allergies could not be determined. Finally, the clinical appropriateness of each perioperative regimen was not directly evaluated at the individual patient level, as the labeled antibiotics included agents other than cefazolin.

In conclusion, antibiotic allergy labels in the EHR were often incomplete and infrequently assessed, partly due to the avoidance of first-line prophylaxis in a substantial proportion of the cohort. Improving the design of EHR to enable allergies to be distinguished from nonallergic adverse events; standardizing the documentation format; and implementing a clinically workable assessment pathway may increase appropriate cefazolin use, advance antimicrobial stewardship, and potentially improve patient outcomes.
